# Supplementary figures and images for: An anti-Gn glycoprotein antibody from a convalescent patient potently inhibits the infection of severe fever with thrombocytopenia syndrome virus
Source: PLoS Pathog. 2019 Feb 1;15(2):e1007375. doi: 10.1371/journal.ppat.1007375 (PMC6380599; doi:10.1371/journal.ppat.1007375)

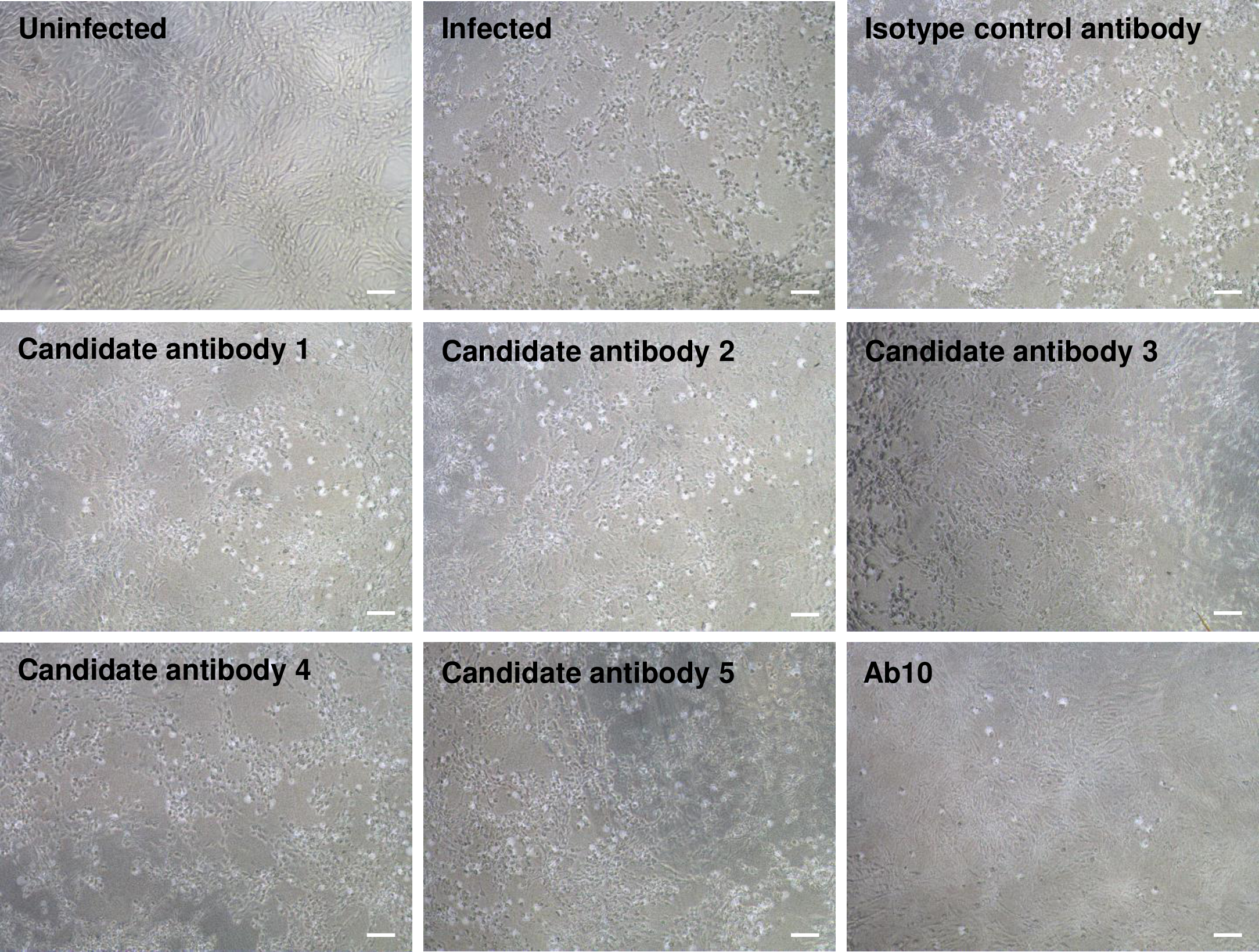

Supplement: S1 Fig — The cytopathic effects (CPE) of SFTSV on Vero cells were monitored to evaluate the protective effect of antibody clones. Candidate antibodies (scFv-Fc format) were mixed with 100 TCID50 of SFTSV (strain: Gangwon/Korea/2012) at a final concentration of 50 μg/mL and the mixtures were incubated for 1 h. SFTSV-antibody mixtures were then transferred to Vero cells at 80% confluency grown in 96-well tissue culture plates, and were incubated for 1 h. Then, cells were washed with PBS and incubated with fresh growth medium for 96 h. Cells were observed under a microscope to evaluate CPE and brightfield images are shown (scale bar: 100 μm). In the control groups, cells not incubated with virus (Uninfected), cells infected without antibody treatment (Infected), cells incubated with virus, and the isotype control antibody (Isotype control antibody) were employed. (TIF) [file ppat.1007375.s001.tif]

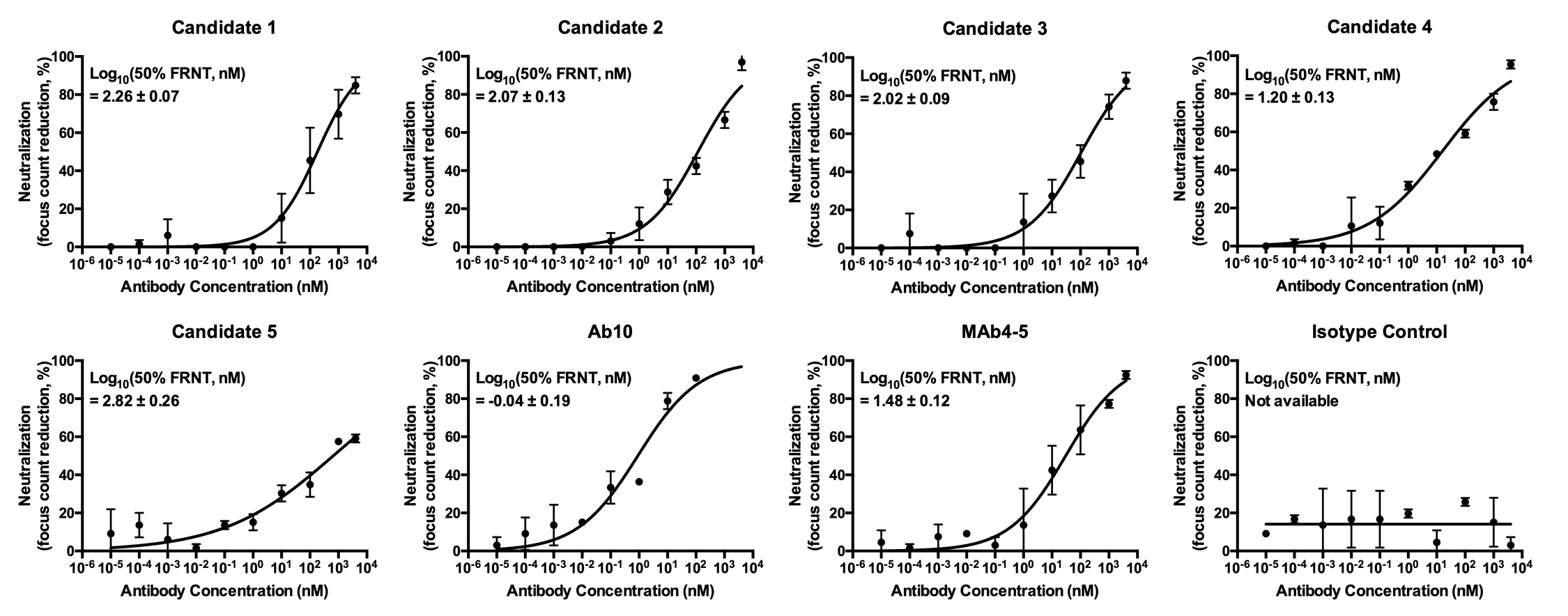

Supplement: S2 Fig — Thirty to fifty focus forming units (FFU) of SFTSV were incubated with serially diluted scFv-Fc fusion proteins for 1 h at room temperature and transferred to Vero cells in a 24-well tissue culture plate. After incubation for 1 h at 37°C in a 5% CO2 incubator, the cells were overlaid with 0.5% methylcellulose in RPMI medium with 2% fetal bovine serum and cultured for 2 days. Cells were fixed with ice-cold methanol for 15 min and incubated with 1% bovine serum albumin in PBS for 1 h. Then, SFTSV localized clusters (foci) were visualized by incubating with 1 μg/mL of anti-SFTSV Gc glycoprotein antibody (Clone Ab3 from patent PCT/KR2017/003156) for 1 h, followed by incubation with 1:2,000 diluted goat anti-rabbit IgG Fc fragment specific antibody, conjugated with HRP (111-035-008; Jackson ImmunoResearch, West Grove, PA, USA) for 30 min and DAB substrate (K5007-BC; Dako). The percentage of neutralization was calculated for each diluted solution of antibody as the percentage of decreased fraction in the number of foci compared to that of the virus without incubation of scFv-Fc fusion protein. An irrelevant scFv-Fc fusion protein was used as an isotype control. Dose-response curves were drawn by non-linear regression analyses (variable slope model) and 50% FRNT values were determined from graphs using GraphPad Prism6 software. (TIF) [file ppat.1007375.s002.tif]

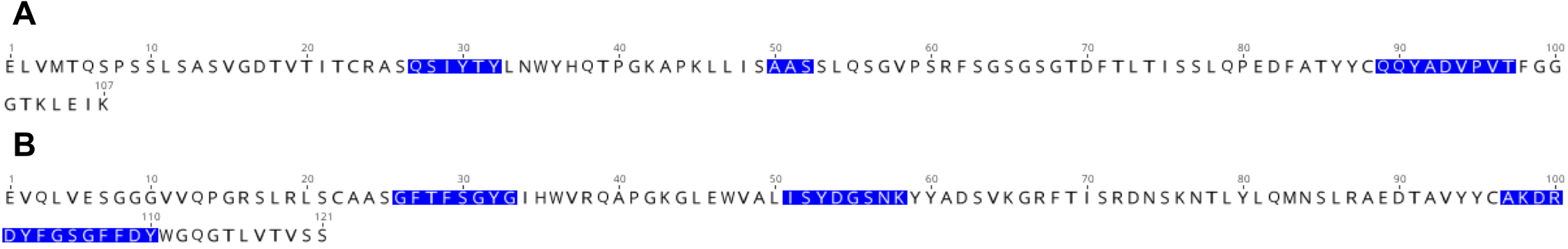

Supplement: S3 Fig — The amino acid sequence of the light chain variable region (A) and heavy chain variable region (B) are shown. Blue letters indicate complementary determining regions (CDR) of each variable region defined by the International Immunogenetics Information System (IMGT). (TIF) [file ppat.1007375.s003.tif]

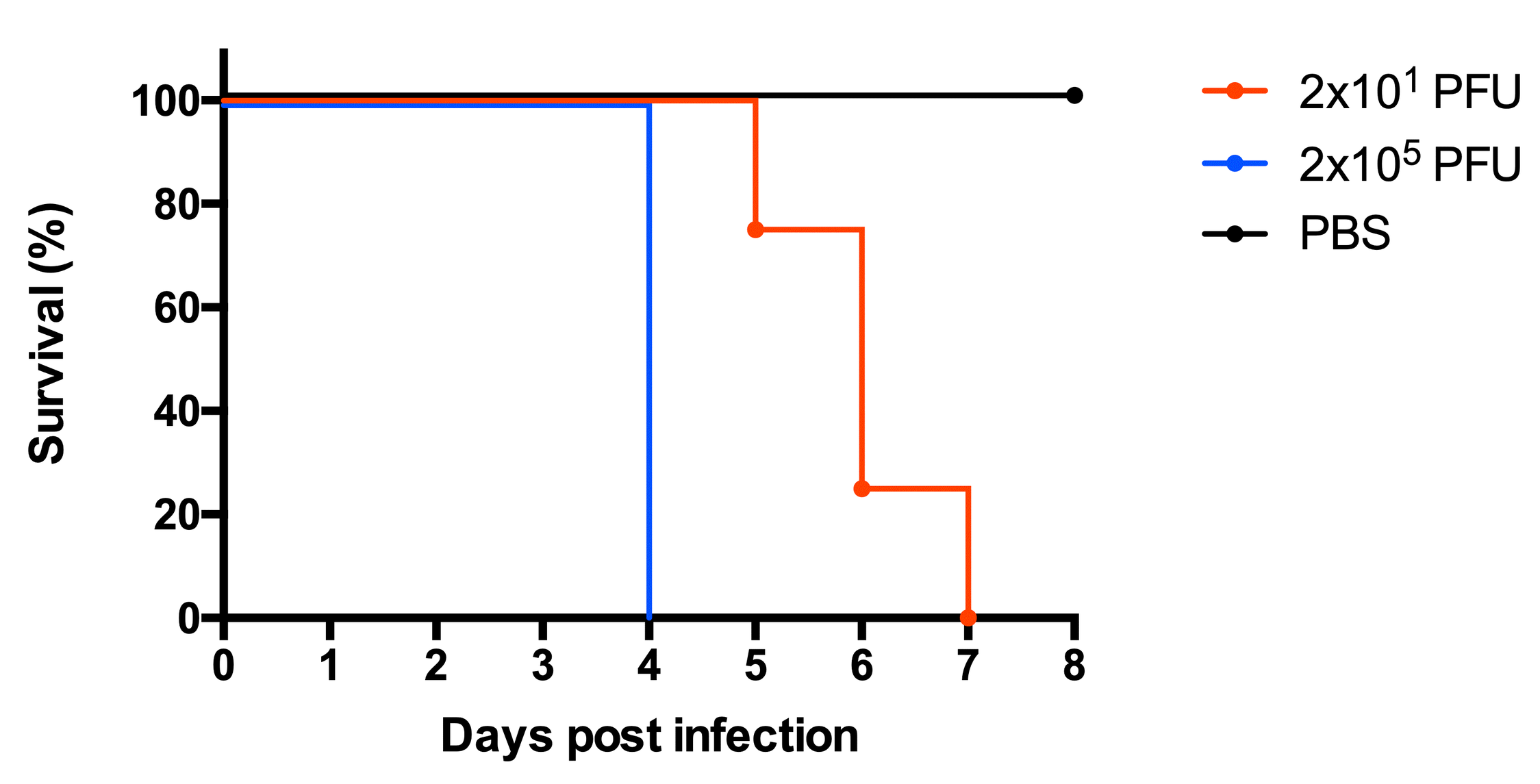

Supplement: S4 Fig — The 8-week-old A129 mice (n = 4 per group) were inoculated with 2×10^5^ or 2×101 PFU of SFTSV (strain: Gangwon/Korea/2012) or PBS vehicle control using a subcutaneous route. The percentage survival was monitored daily until 8 days post-infection. Survival was determined by the Kaplan-Meier method. (TIF) [file ppat.1007375.s004.tif]

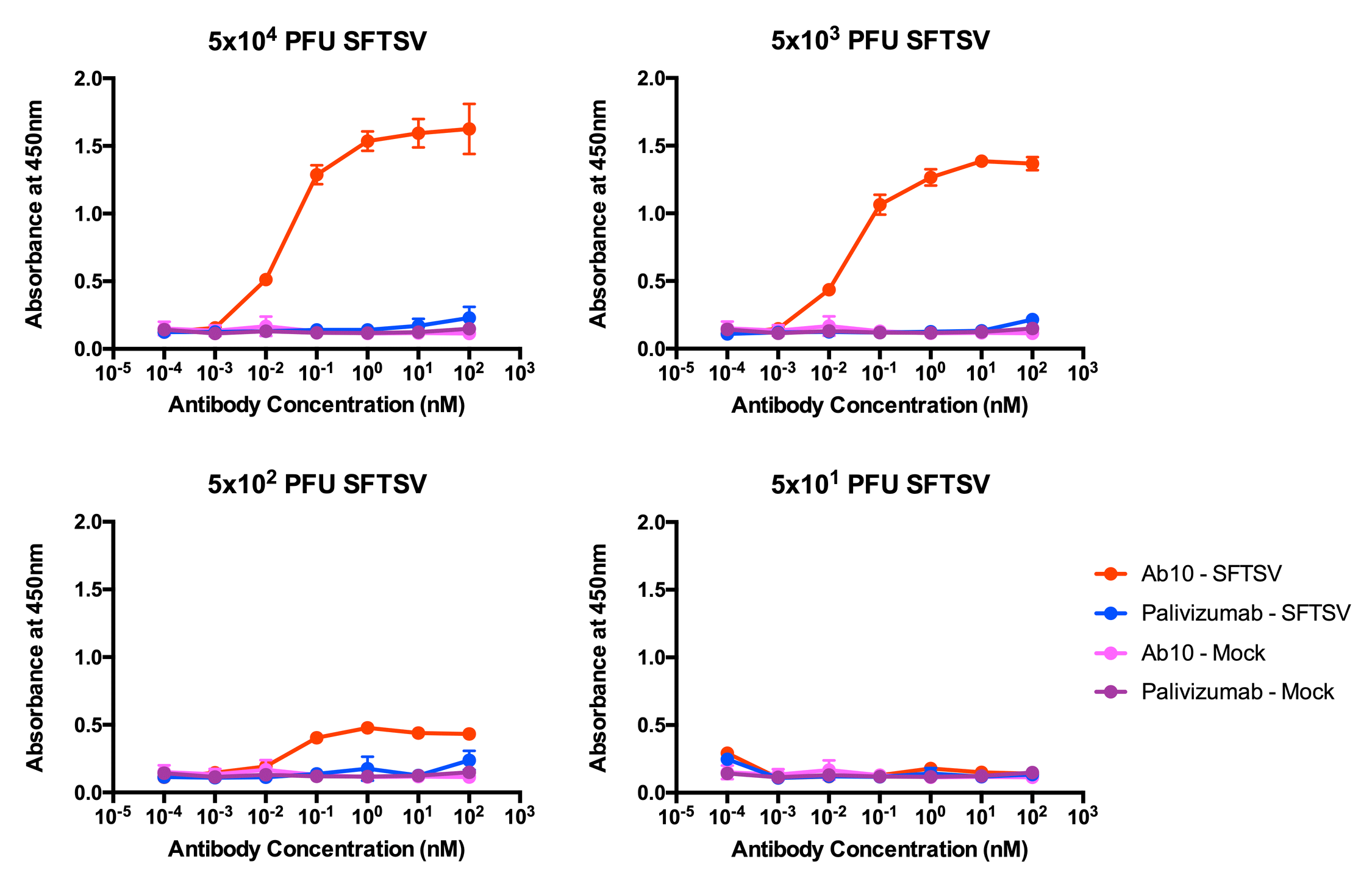

Supplement: S5 Fig — To examine binding activity of Ab10 antibody to SFTSV generated from Vero cells, serially diluted viral supernatants of SFTSV infected cells with a determined titer or the supernatant of mock-infected cells was coated onto microtiter plates (2692; Costar) at 4°C overnight. Fifty to five thousand PFU of SFTSV were used to coat each well. The plates were then incubated with serial dilutions of Ab10 antibody or Palivizumab as an isotype control, followed by HRP-conjugated anti-human IgG Fc antibody (31423; Invitrogen). Reactions were developed by adding TMB substrate (34028; Thermo Scientific) and were terminated by adding 2 M sulfuric acid. The absorbance was measured at 450 nm. The amount of virus coated on each microplate well is indicated on the top of each graph, and the mean absorbance with standard deviation (s.d.) error bars is shown for each antibody concentration. Absorbance of Ab10 antibody bound to SFTSV-coated wells (red), Palivizumab bound to SFTSV-coated wells (blue), Ab10 antibody bound to mock-virus coated wells (magenta), and Palivizumab bound to mock-virus coated wells (purple) are shown in the graph. (TIF) [file ppat.1007375.s005.tif]

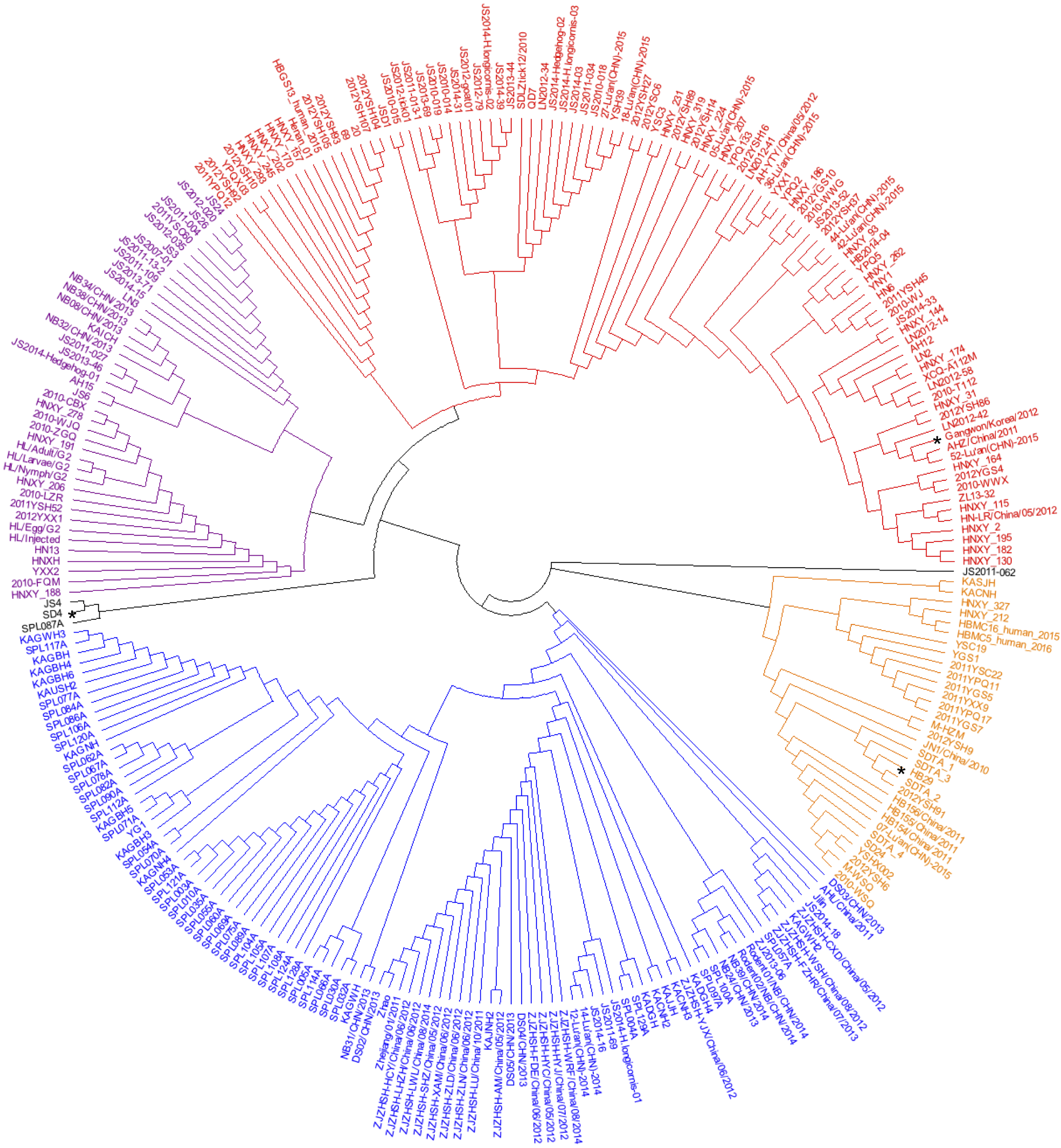

Supplement: S6 Fig — The amino acid sequence of Gn glycoprotein from 272 SFTSV isolates deposited in ViPR were used for analysis. The sequences were trimmed to retain the amino acid residues from 20–452 that corresponded to the ectodomain. Trimmed sequences were analyzed, and a phylogenetic tree was built in a circular tree layout using the neighbor-joining method with a Jukes-Cantor genetic distance model. The names of isolates are labeled beside the tip of each branch. Asterisks at the tip of branches indicate the isolates that were tested for binding activity of Ab10. (TIF) [file ppat.1007375.s006.tif]

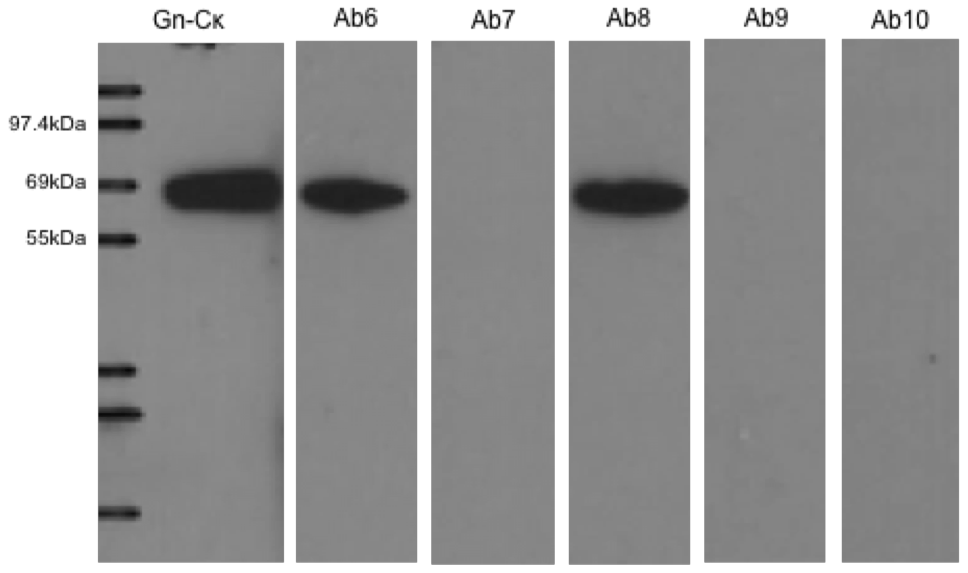

Supplement: S7 Fig — Recombinant SFTSV Gn-Cκ was prepared with sample buffer and reducing agent (NP0008 and NP0004; Invitrogen). The samples were then separated on a polyacrylamide gel (NP0321BOX; Invitrogen) by electrophoresis and transferred to a nitrocellulose membrane. After blocking with 5% (w/v) skim milk in Tris-buffered saline (pH 7.4) the membrane was incubated with 100 ng/mL of five (Ab6 to Ab10) SFTSV Gn specific antibodies in a scFv-Fc format. Gn bound antibodies were probed with HRP-conjugated anti-human IgG Fc antibody (31423; Invitrogen). To confirm the presence of Gn-Cκ protein, HRP-conjugated anti-human Ig kappa light chain antibody (AP502P, Chemicon, Temecula, CA, USA) was used to directly detect Gn-Cκ. The blots were visualized using a chemiluminescent substrate (34578; Thermo Scientific). (TIF) [file ppat.1007375.s007.tif]

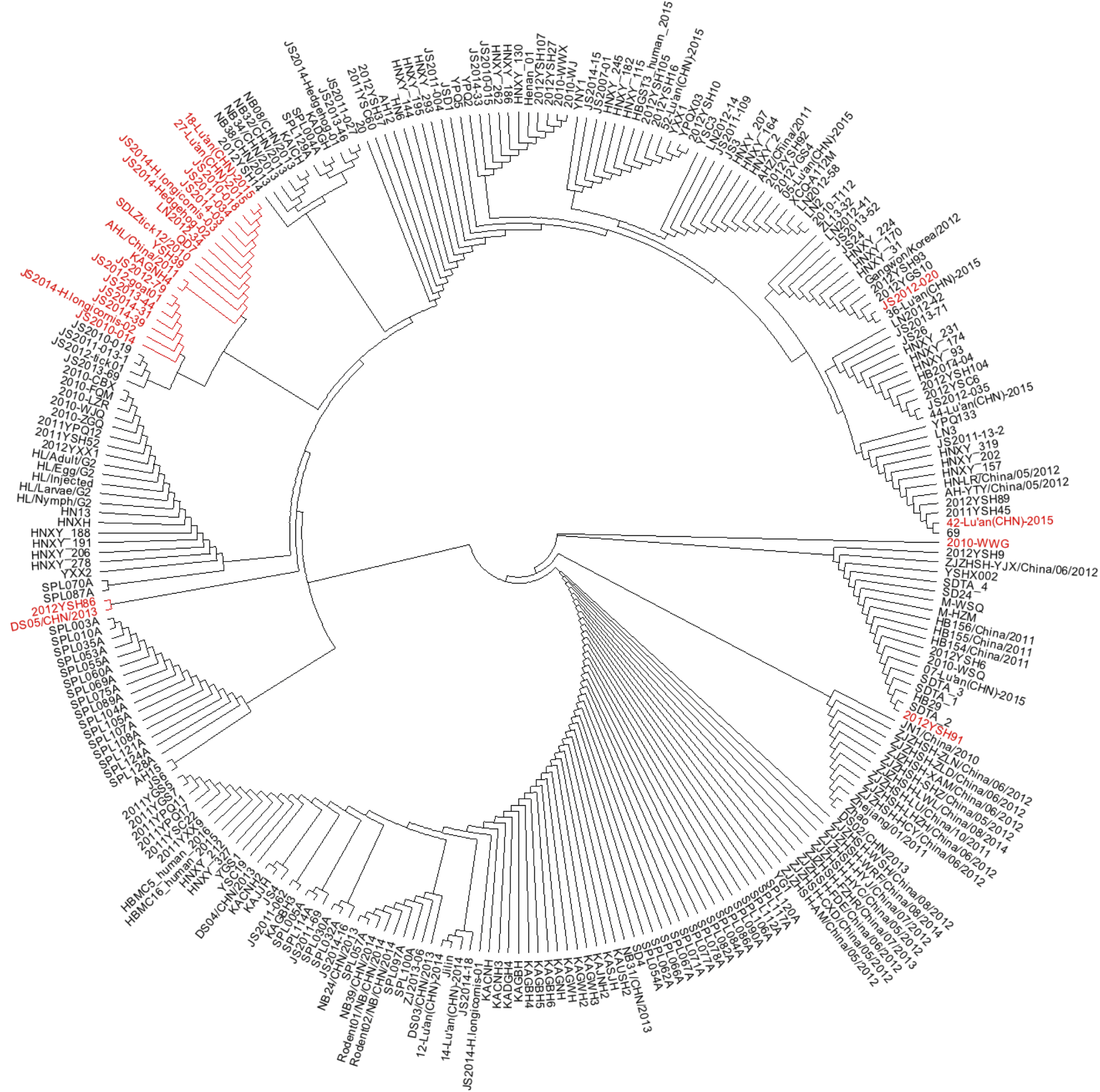

Supplement: S8 Fig — The amino acid sequence of Gn glycoprotein from the 272 SFTSV isolates deposited in ViPR were analyzed. The sequences were trimmed to retain the amino acids from 313–389 that correspond to the residues recognized by Ab10. Trimmed sequences were analyzed, and a phylogenetic tree was built in a circular tree layout using the neighbor-joining method with a Jukes-Cantor genetic distance model. The names of isolates are written beside the tip of each branch. Strain names labeled in red indicate that the Gn glycoprotein of the indicated strain is predicted to not interact with Ab10. (TIF) [file ppat.1007375.s008.tif]
